# Ultrasound Examination of Common Carotid Adventitial Thickness Can Differentiate Takayasu Arteritis and Large Vessel Giant Cell Arteritis

**DOI:** 10.3390/jpm14060627

**Published:** 2024-06-12

**Authors:** Pierluigi Macchioni, Giuseppe Germanò, Nicolò Girolimetto, Giulia Klinowski, Letizia Gavioli, Francesco Muratore, Alessia Laneri, Caterina Ricordi, Chiara Marvisi, Luca Magnani, Carlo Salvarani

**Affiliations:** 1 Division of Rheumatology, Arcispedale Santa Maria Nuova, IRCCS, 42123 Reggio Emilia, Italy; giuseppe.germano@ausl.re.it (G.G.); nicolo.girolimetto@ausl.re.it (N.G.); giulia.klinowski@ausl.re.it (G.K.); letizia.gavioli@ausl.re.it (L.G.); francesco.muratore@ausl.re.it (F.M.); alessia.laneri@ausl.re.it (A.L.); chiara.marvisi@ausl.re.it (C.M.); luca.magnani@ausl.re.it (L.M.); carlo.salvarani@ausl.re.it (C.S.); 2 Department of Surgery, Medicine, Dentistry and Morphological Sciences with Interest in Transplant, Oncology and Regenerative Medicine, University of Modena and Reggio Emilia, 41124 Modena, Italy

**Keywords:** Takayasu arteritis, large-vessel giant cell arteritis, adventitial layer, ultrasound

## Abstract

Pathological studies have demonstrated that the adventitial layer is markedly thickened in Takayasu (TAK) as compared to large vessel giant cell arteritis (LV-GCA). An ultrasound (US) examination of the arterial vessels allows the determination of intima media thickness (IMT) and of adventitial layer thickness (extra media thickness (EMT)). No previous study has evaluated if there are differences in EMT thickness between TAK and LV-GCA. In this cross-sectional retrospective study of stored ultrasound (US) imaging, we have compared common carotid artery (CCA) EMT and IMT in a series of consecutive TAK and LV-GCA patients. US examination CCA IMT and EMT were significantly higher in TAK as compared to LV-GCA. With ROC curve analysis, we have found that an EMT > 0.76 mm has high sensitivity and specificity for TAK CCA examination. The percentage of CCA at EMT > 0.76 mm and the total arterial wall thickening were significantly higher in TAK group examinations. EMT thickness correlated with disease duration and IMT in the TAK group, as well as with the IMT and ESR values in the LV-GCA group. Upon multivariate logistic regression analysis, factors independently associated with TAK CCA were EMT > 0.76 mm and age. No significant variation in IMT and EMT could be demonstrated in subsequent US CCA examinations.

## 1. Introduction

The most common forms of large vessel vasculitis (LVV) include Takayasu arteritis (TAK) and giant cell arteritis (GCA) [1,2]. Imaging studies have demonstrated large vessel involvement of cranial GCA up to 70% of cases mainly localized at aorta, its major branches and axillary arteries, so-called large vessel GCA (LV-GCA) and others (about 10%) may presents only large vessels involvement without cranial artery inflammation [1,2,3]. 

In a recent study of 133 GCA patients, the US evaluation of their peripheral arteries demonstrated that only 30.0% of patients had isolated cranial GCA, 15.8% had isolated LV-GCA, and 54.1% had mixed GCA [4] 

TAK and LV-GCA may be the spectrum of the same disease because they have common pathological alterations characterized by inflammatory changes involving all the three layers of arterial wall [5,6]. 

However, the evolution of the acute inflammatory phase is different between the two diseases, leading to a more marked adventitial fibrosis in the TAK arterial vessels and consequently a more frequent evolution in arterial stenosis than in their LV-GCA counterpart [7]. 

Pathological studies reported that aortic adventitial expansion helps in separating TAK from LV-GCA aortitis [8,9]. 

EULAR recommendations and OMERACT definitions for the diagnosis and follow-up of patients with LVV consider the US-measured IMT of superficial temporal arteries and large extra-cranial vessels above some reference values the most specific and sensitive parameter for diagnosis and follow-up of patients affected by LVV because of its high sensitivity and specificity as diagnostic tool in specific clinical settings and because of its sensitivity to change during treatment [10,11,12,13,14,15,16,17].

No mention is made about arterial adventitial layer. However, recent works have outlined the usefulness of common carotid artery (CCA) extra IMT measurement (EMT) of patients with atherosclerosis for its higher predictive value of clinical ischemic complications as compared to the measurement of IMT alone [18,19,20].

Moreover, older and more recent studies have recognized the prominent role of adventitial resident cells and adventitial vessels as initiating factors of vessel wall inflammation [9,21,22,23,24,25].

US examination of the CCA may differentiate IMT from adventitial layer because of the different acoustic signal and it is demonstrated as a reliability tool to measure EMT [18].

No previous study has evaluated CCA EMT in LVV. The aims of this study are to compare the IMT and EMT of the CCA of LVV patients being followed up in a tertiary rheumatological center, to evaluate its usefulness in differentiating TAK vs. LV-GCA, examine demographic, clinical, and imaging factors associated with EMT, and elucidate the relationship between these variables. Moreover, we examine its variation during follow-up. 

## 2. Materials and Methods

This is a retrospective study of a consecutive series of LVV patients (TAK and LV-GCA) being followed up at our tertiary center at Arcispedale S. Maria Nuova of Reggio Emilia, Reggio Emilia, Italy. 

Between 1 January 2012 and 31 December 2023, the consecutive LV-GCA and TAK patients who had been referred to the Rheumatology Unit at the Arcispedale S. Maria Nuova in Reggio Emilia in Italy, and who had stored imaging of CCA US examinations, were re-evaluated. GCA was diagnosed according to the ACR1990 criteria [26] and LV-GCA in the presence of LVV involvement, as observed from their CT and/or MRI and/or ultrasonography and/or PET examinations, according to GIACTA criteria [27]. 

TAK arteritis was diagnosed using a set of diagnostic criteria (Ishikawa’s diagnostic criteria [28] and its modification by Sharma et al. [29]) and ACR classification criteria for TAK [30]. 

The diagnosis of LV-GCA and TAK was confirmed by an imaging technique and defined as the presence of circumferential wall thickening/wall edema with or without contrast enhancement and/or the presence of vascular stenosis/occlusion and/or vascular dilatation/aneurysm on US examination, computed tomography angiography (CTA) or magnetic resonance angiography (MRA). Imaging features consistent with the presence of LVV were considered one of the following: the presence of long segments of smooth arterial stenosis or smooth tapered occlusion and/or vascular dilatation/aneurysm on angiography; the presence of vascular 18F-fluorodeoxyglucose (18F-FDG) uptake (>2 according to Meller on FDG–positron-emission tomography (FDG-PET) [31]; and the presence of a hypo-isoechoic circumferential wall thickening not attributed to atherosclerotic changes on US. 

As a tertiary reference center for vasculitis, we observed patients with suspected, early, or established LVV for the re-evaluation of treatment and/or definition of disease activity. Patients are evaluated with a standardized imaging screening upon the first visit and yearly for a comprehensive evaluation of arterial disease activity and extension using US, CTA, or MRA, and/or 18F-FDG-PET/CT. 

Imaging studies at diagnosis and during the follow-up of each patient were evaluated by a radiologist and a nuclear medicine physician. All US examinations were performed by three operators (GG, PM, NG) expert in vascular US.

This protocol also included the determination of the erythrocyte sedimentation rate (ESR) (Westergren method) and C-reactive protein (CRP) serum levels (nephelometric method) at the time of diagnosis, control visits, and imaging evaluation.

Patient consent was waived due to the retrospective nature of the study not involving patients but only their stored US images. All medical records of these patients were reviewed. Aside from the demographic features, the following clinical data at the time of diagnosis were assessed: headache, abnormal temporal arteries on physical examination, scalp tenderness, jaw claudication, carotidynia, visual manifestations (transient visual loss including amaurosis fugax, permanent visual loss, and diplopia), cerebrovascular accidents (CVAs) (stroke and/or transient ischemic attack), findings of vascular ischemia (claudication, diminished or absent pulse, bruit, vascular pain), systemic signs/symptoms (at least one of the following: fatigue, anorexia, weight loss of at least 4 kg, or fever), fever > 38 °C, polymyalgia rheumatica (PMR) (bilateral marked aching and stiffness without other apparent cause in at least two of the following regions: neck, shoulder girdle, and hip girdle), and distal musculoskeletal manifestations. ESR was determined using the Westergren method (since most of our patients were women over the age of 50, the upper limit of normal considered for ESR was set at 30 mm/h). CRP was measured by nephelometry (NA latex CRP kit; Behringwerke, Marburg, Germany; upper limit of the normal reference ranges 0.5 mg/dL).

All patients were initially treated with prednisone (PD) for a variable time according to treatment response or adverse events (AE). Immunosuppressive (methotrexate, azathioprine, mycophenolate mofetil, cyclophosphamide) and/or biological agents (tocilizumab, TNF inhibitors, rituximab) were used according to the judgment of the doctor who treated the patient. 

Complete clinical examination, determination of laboratory parameters (including acute-phase reactants), imaging of the epi-aortic vessels, aorta, and peripheral arteries (when indicated) were used to evaluate disease activity according to the criteria of Kerr et al. [32]. However, to compute Kerr’s index, we used mostly morphologic imaging techniques instead of digital subtraction angiography. Kerr’s index > 1 definition of an active disease and treatment was changed accordingly.

### 2.1. Carotid US Examination

All patients had a standard CDUS examination of CCA using a high-frequency linear probe (EsaoteMyLab70 or EsaoteMyLabClassC, 13–5 MHz linear array transducer, Esaote SpA, Genoa, Italy). Stored US imaging of 258 exams were reviewed by one of the experts (PM) and intima-media thickness (IMT) was measured at the far wall of the right CCA, proximally to the bifurcation, and its highest values (outside the atherosclerotic plaques) were recorded. CCA extra media thickness (EMT) was assessed at the site of the IMT measurement as the thickness of the hyperechoic structure between the hypo-anechoic media and the surrounding hypo-anechoic perivascular tissue, according to the literature [18,33]. This measure may be considered a valid surrogate of the real adventitial thickening. A hypoechoic, circumferential, homogeneous IMT > 1.0 mm was considered consistent with active vascular involvement of the vessels and the presence of hypo/hyperechoic non-homogeneous IMT > 1.0 mm a sign of chronic vessel involvement. A CCA with IMT > 1.0 mm was considered involved in the vascular inflammation, while a CCA with IMT < 1.0 mm was not involved.

Non-homogeneous, eccentric, partly calcified arterial wall thickenings were considered consistent with atheromatosis. A carotid plaque is defined as a focal structure that protrudes into the arterial lumen and is of at least 0.5 mm or 50% of the surrounding IMT value, or that demonstrates a thickness >1.5 mm measured from the media-adventitia interface to the intima-lumen interface [34].

### 2.2. Statistical Analysis

Continuous data were described as mean and standard deviation (mean + SD) or median and interquartile range (IQR), and categorical variables as absolute frequencies and percentages. Continuous variables were compared by using Student’s *t*-test or the Mann–Whitney test when the distributions were skewed. Comparison of categorical variables was performed using the Chi Squared or Fisher’s exact test. Correlations between the variables were evaluated by Spearman’s rho. To evaluate the prognostic performance of EMT, a receiver operating characteristic (ROC) curve was constructed for discrimination between the TAK and LV-GCA examinations. The area under the ROC curve (AUC) values provided a measure of the overall discriminative ability of the parameter. The ROC area and its standard error were estimated using a non-parametric approach. We used Youden’s index to determine the cut-off value of EMT to identify TAK patients. Clinical and ultrasonographic variables with a *p* value < 0.1 in discriminating TAK and LV-GCA examinations, and EMT values higher or lesser than the value obtained by ROC analysis (0.76 mm), entered as possible explanatory variables in a multivariate logistic regression analysis using a backward selection procedure. The most significant independent variables were identified using a *p* value greater than 0.10 as the removal criterion. To estimate the intra-rater reproducibility of EMT and IMT values, a double assessment of 50 random stored pictures of CCA artery walls was carried out 4 months apart. Intra-rater agreement analyses were evaluated by means of the intraclass correlation coefficient (ICC). All tests were two-sided; significance was set at *p* < 0.05. Statistical analysis was performed using SPSS version 28.0 (IBM Statistics, Armonk, NY, USA; IBM Corp, USA). 

## 3. Results

A total of 261 stored US images of the most involved CCA (123 exams of 72 TAK and 138 of 81 LV-GCA patients) were reviewed for IMT and EMT measurements. Fifty exams (19.2%) did not have images useful for EMT measurement and were not considered for the study. Up to 120 patients (55 TAK and 65 LV-GCA) out of a total of 211 CCA US examinations (103 in the TAK group and 108 in the LV-GCA group) were evaluated for the study. Furthermore, 61 patients had one examination and 59 had two or more US examinations. Subsequent US examinations were performed at a mean distance of 22.5 ± 3.2 months. 

Table 1 reports demographic and clinical cumulative data of the patients at the time of every US examination.

Percentage of female and disease duration was significantly higher in the TAK group, while age was significantly lower as compared to the LV-GCA group (*p* < 0.001 for the two comparisons). There were no statistical differences in the percentage of patients taking steroids (CS) at the time of the US examination, while the percentage of patients in methotrexate (MTX) treatment was higher in TAK group (*p* = 0.020). The distribution of biological (BIO) drugs was different in the two groups, with a higher percentage of tocilizumab (TCZ) treatment in TAKA and higher tumor necrosis factors inhibitor (TNFi) in the LV-GCA group (*p* < 0.001). The durations of CS treatment and BIO therapy were significantly longer in the TAK group (*p* = 0.003 and *p* < 0.001, respectively). There were no differences in Kerr’s activity score while ESR and CRP were significantly higher in the LV-GCA group (*p* < 0.001 for both comparisons).

The US examinations of the CCA showed that atherosclerotic plaques were significantly more prevalent in the LV-GCA group (*p* < 0.001). IMT and EMT were significantly higher in the TAK group (*p* = 0.002 and *p* < 0.001 respectively) as compared to LV-GCA, and the same result was observed for a percentage of patients with IMT > 1.0 mm (*p* = 0.011) and of the patients with EMT > 0.76 mm (*p* < 0.001). As a consequence, total arterial wall thickening and the ratio of IMT/EIMT were significantly higher in the TAK group (*p* < 0.001 for the two comparisons) (Table 2).

In the bivariate analysis, correlation coefficients (spearman’s rho) between EMT and IMT had a similar statistical significance in the TAK and LV-GCA examinations (0.204 (*p* = 0.044) and 0.289 (*p* = 0.003), respectively). Correlation coefficients between EMT and disease duration were significant only in the TAK group, while correlation coefficients with ESR was significant only in the LV-GCA group (Table 3). 

We plotted an ROC curve for EMT discriminative ability for the TAK vs. LV-GCA groups, obtaining an ROC AUC of 0.848 (95% CI 0.78–0-94) with a significant asymptotic *p* < 0.001. An EMT value of 0.76 mm has a sensitivity of 66.02% (95% CI 56.0–75.0) and a specificity of 89.8% (95% CI 82.5–94.8, a LR+ 6.48 (95% CI 3.64–11.54), an LR- 0.38 (95% CI 0.29–0.50), a PPV 86.1 (95% CI 77.6–91.7) and an NPV 73.5 (95% CI 67.8–78.5) in correctly classifying a TAK patient’s examination. When only the examinations with CCA IMT > 1.0 mm were considered, the results were almost the same as with an ROC AUC = 0.874 (95% CI 0.80–0.94), asymptotic *p* < 0.001, and Youden’s index cut-off value of 0.76. 

As expected, an EMT measure > 0.76 mm was more prevalent in the TAK group as compared to the LV-GCA group (66% vs. 10.2%, *p* < 0.001, OR 17.1 (95%CI 8.13–36.1). IMT was higher in EMT > 0.76 vs. < 0.76 (*p* < 0.001) with higher prevalence of IMT > 1.0 mm (*p* < 0.001) in the EMT > 0.76 group. It was more prevalent in female sex (*p* < 0.001), and EMT examinations with EMT > 0.76 have a longer disease duration (*p* < 0.001), were older (*p* = 0.007), have a longer bio-treatment duration (*p* = 0.016), and lower CRP serum values (*p* = 0.008).

Then, EMT value > 0.76 vs. <0.76 was examined separately in the two groups. In the TAK group, only IMT measure and IMT prevalence > 1.0 mm maintained significance (*p* = 0.007 and *p* = 0.005, respectively), while in the LV-GCA group, prevalence of atheromatic plaque was higher in EMT > 0.76 (2.1% vs. 36.4%, *p* < 0.001) with a significant lower serum CRP value (p = 0.008).

In a univariate logistic regression analysis of all the cases (Table 4), factors independently associated with the TAK patient’s CCA vs LV-GCA examinations were age, female gender, ESR > 40 mm/first hour, serum CRP > 0.50 mg/dl, IMT > 1.0 mm, and EMT > 0.76 mm. A multivariate logistic regression analysis of only the age and EMT > 0.76 mm maintained significance (*p* < 0.001 and *p* = 0.008, respectively, OR (95% CI) 0.742 (0.66–0.84) and 13.9 (1.98–97.5), respectively).

In multiple logistic regression analysis of all cases, factors independently associated with the presence of an EMT measure > 0.76 mm were IMT > 1.0 mm (OR 3.19, 95% CI 11.47–8.92) and the diagnosis of TAK (OR 8.91, 95%CI 3.98-20.0). In the TAK group cases, factors associated with EMT > 76 mm were IMT > 1.0 mm (OR 5.31, 95%CI 1.89–14.9), while in the LV-GCA evaluations, no factors maintained significance. The variables of treatment, age, sex, and disease duration have no statistical significance in the total group analysis nor in TAK or LV-GCA groups individually.

We then analyzed the variation in CCA US IMT and EMT in two subsequent evaluations in the total group of US examinations, and then separately for the TAK and LV-GCA groups (Table 5). 

Paired evaluations were available for 75 cases in the total group, 39 in the LV-GCA group, and 36 in TAK group. No significant variation in the IMT, EMT, and total vessel wall thickness could be demonstrated with a paired *t*-test in the total group and in the TAK and LV-GCA groups when examined separately. During the second evaluation, 58% of the TAK patients and 60% of the LV-GCA patients had an US reduction in IMT (*p* = 0.839) and 46% and 53% reduction in EMT (*p* = 0.566). 

In the TAK group, at the second evaluation, 4/30 (13.3%) cases with baseline EMT > 0.76 mm had reduced EMT under the 0.76 mm limit, while 3/9 (33.3%) cases with baseline EMT < 0.76 mm had increased values above the 0.76 mm limit. In the LV-GCA group, 1/32 (3.1%) of the group with a baseline EMT < 0.76 mm had increased EMT values above 0.76 mm and 2/4 cases with a baseline value > 0.76 mm decreased to below this limit. 

In multivariate logistic regression analysis of the cases on a whole, a baseline EMT > 0.76 mm was the only baseline variable significantly associated with EMT > 0.76 mm at the second examination (OR 65.1, 95% CI 12.4–261.6). In the TAK group, the result was the same (OR 34.5, 95% CI 3.2–268.5). In the LV-GCA group, the analysis was impossible because of the small number of case variation.

### Agreement of EMT and IMT Measurements 

Excellent intra-rater agreement was observed. The ICC (Cronbach’s alpha) for the two ratings was 0.990 (95% CI 0.983–0.994) for IMT and 0.962 (95% CI 0.935–0.978) for EMT.

## 4. Discussion

In this study, we demonstrated that at US examinations of the CCA IMT and EMT were significantly higher in the TAK group as compared to those of LV-GCA. With the ROC curve analysis, we have found that an EMT measure > 0.76 mm has high sensitivity and specificity for TAK CCA examination. The percentage of CCA IMT > 1.0 mm and CCA EMT > 0.76 mm, and the total arterial wall thickening, were significantly higher in the TAK group examinations. EMT was correlated with disease duration and IMT in the TAK group, while it was correlated with the IMT and ESR values in the LV-GCA group.

Upon multivariate logistic regression analysis, factors independently associated with the TAK group examinations were EMT > 0.76 mm and age. 

We did not find any statistically significant variations in the multiple IMT and EMT determinations of our series. The persistence of US IMT thickness in LVV during treatment is a well-known characteristic of these types of diseases. Moreover, our patients were often seen during the active phase of their disease, with the corresponding IMT higher than during the remission period. We can hypothesize that EMT follows the same direction during active and remission phases of vasculitis. Moreover, the influence of treatment on EMT has never been evaluated in prospective studies. In the TAK group, there was a weak significant correlation between the EMT and disease duration, possibly reflecting the observed tendency of exuberant adventitial fibrosis described in the aortic specimen of this disease. 

Older and more recent studies have reported that TAK aortitis is often associated with a thick aortic wall, a fibrotic rind-like adventitia, and intense medial and adventitial inflammation with micro-abscesses or necrotizing granulomas. At gross appearance, the aorta is thick and often rigid, secondary to fibrosis of all three arterial layers and, particularly, the adventitia and intima. Extension of the adventitial fibrosis and round cell infiltration to the adjacent structures may imitate retroperitoneal fibrosis [35,36].

Miller et al. described TAK aortitis to be characterized microscopically by a fibrous expansion of the adventitia and media layers, and by inflammatory infiltrates of lymphocytes, plasma cells, neutrophils, histiocytes, and rare eosinophils. As a result, patients with Takayasu arteritis have thicker aortic walls than any other form of aortitis studied in papers comprising LV-GCA [37]. 

Watanabe et al. [9] reported that distinguishing findings between TAK and GCA aortitis concentrate mostly on the adventitial layer. In cases of TAK, the adventitia is typically, and often massively, expanded. The disease-induced neotissue is identified as a collagen-rich tissue with fibrotic reactions involving all three layers, with the adventitia being the most involved by far. 

In contrast, the pathological features of GCA aortitis have prominent inner-half involvement compared to the outer half, being relatively sparing within the adventitial layer [8]. 

Previous studies have analyzed immunological cellular activation and cytokines production in LVV [38,39,40], and others have studied the cause of the preponderance fibrous adventitial reaction in TAK patients. IL-6 production [41], activation of monocyte/macrophage lineage [42,43,44,45], high presence of activated mast cells [46], activated neutrophils [47], and the presence of Th17 lymphocyte populations [48,49] all seem to contribute to fibroblast activation and collagen deposition in the adventitial layer. A significant difference between LV-GCA and TAK lies in the composition of the wall-infiltrating immune cell compartment, which in TAK, includes a significant population of CD8+ T cells, a low ratio of CD4/CD8 cells, as well as a higher proportion of natural killer cells, indicating the differences in the inflammatory effector pathway [39].

USs are widely used for diagnosis and the follow-up of LVV disease and in the most recent EULAR recommendations [11], the use of artery US examinations are considered the first imaging modality in suspected cranial or axillary GCA, and only as an alternative modality after MRI or PET/CT scan in TAK patients and when LV-GCA is suspected. The use of an US is suggested as one of the first-line imaging modalities in cases of suspected relapses and is not routinely recommended in cases of clinical remission. All these US evaluations are centered around IMT US characteristics and measurements because of their high sensitivity and specificity for LVV diagnosis, and for their role as surrogates of treatment response. No mention about EMT measurement is made in the TAK or LV-GCA patient’s evaluations.

Recent works have examined US CCA IMT and EMT measurements in the general population and in patients with vascular disease risk factors. CCA US measurements of IMT and EMT have been reported by Skilton et al. [20] in a study involving 175 subjects, including 54 with diabetes, 43 with dyslipidemia, 26 with other cardiovascular risk factors, and 52 healthy control subjects. When compared with the control subjects, EMT was increased in both the diabetes (*p* = 0.0001) and dyslipidemia (*p* = 0.04) groups. Multivariate linear regression analyses revealed that diabetes, high-density lipoprotein cholesterol (inverse association), and systolic blood pressure were the factors most strongly associated with EMT. These associations seem to be independent of the carotid IMT. In another series of 50 patients with ages ranging from 20 to 79 years, Carlini et al. [50] found that carotid USs determined that CCA EMT was positively correlated with age, blood pressure, and carotid stiffness, and negatively correlated with carotid distensibility. All endpoints correlated with EMT remained consistent after adjusting for BMI or sex. These data suggest CCA EMT as a clinically relevant target that may be associated with age-related CVD risk in humans.

Moreover, two studies of Haberka et al. [51,52] reported the significant correlations among CCA EMT, the presence of a metabolic syndrome, and the presence of cardiac arterial disease (CAD). 

A recent work by Ferreira et al. [53] demonstrated the presence of a significant correlation among US-determined CCA EMT, the presence of carotid plaques, IMT, and the area of the highest plaques.

These data outline the importance of US EMT as a novel vascular index associated with the presence and severity of artery disease [52,53].

There are no US indexes that can predict the structural artery wall alterations observed during TAK disease nor are there any drugs able to interfere with this evolution. Serial examinations during the follow-up of EMT in TAK patients could have a predictive value in recognizing patients with incipient structural damage. Moreover, in LV-GCA and TAK patients, common carotid EMT determination gives the opportunity to recognize the group of patients with higher CVD risk associated with the presence of traditional risk factors.

The most important limitation of this study is its retrospective and transverse nature with patients’ data collected in different phases of the diseases, mainly during relapses, and for the TAK group, almost no data were available at disease onset. The significantly different disease durations between the two groups may interfere with the correctness of our conclusions. Moreover, it was impossible to define the role of treatment on EMT variations.

Cardiovascular risk factors were not routinely collected in our patients. Serum lipids, hypertension, diabetes, smoking, and other factors might have an impact on common carotid EMT thickness. However, we think that they would be more prevalent in the LV-GCA group, and because of their contribution to EMT, our results are even stronger.

In conclusion, we have found, in a consecutive series of LVV patients, that US-measured EMT is higher in the TAK group as compared to the LV-GCA group examinations, that a cut-off value of 0.76 has high sensitivity and specificity for the TAK group examinations, and that higher values of EMT are correlated with disease duration and IMT measurement. Finally, no temporal variations of EMT could be demonstrated in our serial examinations.

We recommend, in future studies, the measurement of EMT to establish its possible role in the diagnosis and follow-up of patients with LVV.

## Figures and Tables

**Table 1:** Clinical and laboratory features of patients at the time of US common carotid artery examinations.

	TOTAL (211 pts)	TAKA (103 pts)	LV-GCA (108 pts)	*p*
Age (y)	53.1 ± 19.4	37.2 ± 11.6	70.1 ± 8.4	<0.001
Female (126 pts) (%)	95/126 (75.4%)	59/62 (95.2%)	36/64 (56.3%)	<0.001
Disease duration (m)	67.7 ± 71.0	100.3 ± 84.9	38.6 ± 44.6	<0.001
Actual steroid treatment	145 (65.3%)	73 (64,6)	72 (65)	1.0
Steroid treatment duration (m)	60 ± 122	58.7 ± 62.4	31.8 ± 37.5	0.003
Actual MTX treatment	38 (20.0%)	26 (26.5%)	12 (13%)	0.020
MTX treatment duration (m)	7.4 ± 8.1	24.6 ± 35.2	12.6 ± 25.2	0.098
Actual BIO treatment	79 (%)	45 (%)	34 (%)	0.086
TNFi/TCZ/RTX	43/34/2 (54%/43%/2.5%)	14/30/1 (31%/67%/2%)	29/4/1 (85%/12%/3%)	<0.001
Bio treatment duration (m)	11.2 ± 20.6	18.0 ± 28.1	6.8 ± 12.4	<0.001
KERR ≥ 2	76 (34.2%)	37 (33.3%)	39 (35.2%)	0.823
ESR (mm/1st hour)	25.6 ± 25.8	21.2 ± 21.8	37.2 ± 39.8	<0.001
CRP mg/dL)	1.95 ± 3.38	0.89 ± 1.39	3.29 ± 5.29	<0.001
ESR > 40 mm/1 h (197)	44 (22.3%)	11 (11%)	33 (34%)	<0.001
CRP > 0.5 mg/dL (198)	83 (41.9%)	35 (35%)	48 (49%)	0.046

**Table 2:** Ultrasound features of common carotid artery examination.

	TOTAL (211)	TAK (103)	LV-GCA (108)	*p*
Atheromasic plaque	49 (22.1%)	9 (1.2%)	40 (36.5%)	<0.001
Common Carotid artery IMT (mm)	1.19 ± 0.64	1.27 ± 0.60	1.05 ± 0.41	0.002
Common Carotid artery IMT > 1.0 mm	129 (53.1%)	72 (61.5%)	57 (45.2%)	0.011
Common carotid artery EMT (mm)	0.79 ± 0.33	0.94 ± 0.37	0.64 ± 0.16	<0.001
EMT > 0.76 mm	85 (40.3%)	68 (66%)	11 (10.2%)	<0.001
Total Arterial Wall Thickening	1.93 + 0.69	2.22 ± 0.75	1.68 ± 0.45	<0.001
Imt/emt thickening RATIO	1.58 ± 0.73	1.37 ± 0.71	1.77 ± 0.94	<0.001

**Table 3:** Correlation coefficient (Spearman’s rho) between variables.

	All pts Rho (*p*)	TAK Rho (*p*)	LV.GCA Rho (*p*)
EMT/age	−0.495 (<0.001)	0.110 (0.275)	−0.120 (0.226)
EMT/ESR	0.069 (0.333)	0.158 (0.116)	0.217 (0.033)
EMT/CRP	−0.076 (0.277)	0.036 (0.724)	0.076 (0.454)
EMT/disease duration	0.393 (<0.001)	0.269 (0.008)	0.074 (0.480)
EMT/PD duration	0.202 (0.025)	0.199 (0.092)	−0.057 (0.694)
EMT/BIO terapy duration	0.192 (0.012)	0.123 (0.264)	0.023 (0.833)
EMT/IMT	0.292 (<0.001)	0.204 (0.044)	0.289 (0.003)

**Table 4:** Factors associated with the TAK vs LV-GCA examinations at mono and multivariate logistic regression analysis.

Variable	Univariate *p*	OR (95% CI)	Multivariate *p*	OR (95% CI)
Age	<0.001	0.745 (0.68–0.82)	<0.001	0.742 (0.66–0.84)
Female gender	<0.001	11.9 (4.51–31.9)	0.515	
ESR > 40 mm	<0.001	0.24 (0.11–0.51)	0.586	
CRP > 0.50	0.047	0.56 (0.32–0.99)	0.344	
IMT > 1.0 mm	0.012	2.04 (1.17–3.57)	0.360	
EMT > 0.76 mm	<0.001	13.5 (8.63–26.1)	0.008	13.9 (1.98–97.5)

**Table 5:** Variations of US measures in two subsequent evaluations.

Variables	T0	T1	*p*
EMT mm (All cases)	0.77 ± 0.34	0.77 ± 0.33	0.928
IMT (mm) (All cases)	1.19 ± 0.45	1.29 ± 1.25	0.536
Vessel Wall thickness (mm) (all cases)	1.96 ± 1.66	1.88 ± 0.64	0.205
EMT (mm) (TAK)	0.97 ± 0.37	0.98 ± 0.36	0.892
IMT (mm) (TAK)	1.41 ± 0.39	1.70 ± 1.65	0.410
Vessel Wall Thickness (mm) (TAK)	2.40 ± 0.55	2.31 ± 0.56	0.261
EMT (mm) (LV-GCA)	0.59 ± 0.15	0.58 ± 0.10	0.746
IMT (mm) (LV-GCA)	1.02 ± 0.43	0.96 ± 0.44	0.416
Vessel Wall Thickness (mm) (LV-GCA)	1.61 ± 0.53	1.55 ± 0.46	0.464

## Data Availability

The datasets presented in this article are not readily available because the data are part of an ongoing study. Requests to access the datasets should be directed to Carlo Salvarani.
